# High prevalence of HIV-1 drug resistance among patients on first-line antiretroviral treatment in Lomé, Togo

**DOI:** 10.1186/1758-2652-14-30

**Published:** 2011-06-10

**Authors:** Anoumou Y Dagnra, Nicole Vidal, Akovi Mensah, Akouda Patassi, Komi Aho, Mounerou Salou, Marjorie Monleau, Mireille Prince-David, Assétina Singo, Palokinam Pitche, Eric Delaporte, Martine Peeters

**Affiliations:** 1 Centre National de Référence des tests VIH-IST/PNLS and Laboratoire BIOLIM, FMMP/UL, Lomé, Togo; 2 UMI 233, Institut de Recherche pour le Développement (IRD) and University of Montpellier 1, Montpellier, France; 3 Espoir Vie Togo (EVT), Lomé, Togo; 4 Service des Maladies infectieuses, University Hospital TOKOIN, Lomé, Togo; 5 Centre de Réflexion et d'Initiative pour la Promotion de la Santé (CRIPS), Lomé, Togo; 6 Programme National de lutte Contre le VIH/SIDA-IST, Lomé, Togo

## Abstract

**Background:**

With widespread use of antiretroviral (ARV) drugs in Africa, one of the major potential challenges is the risk of emergence of ARV drug-resistant HIV strains. Our objective is to evaluate the virological failure and genotypic drug-resistance mutations in patients receiving first-line highly active antiretroviral therapy (HAART) in routine clinics that use the World Health Organization public health approach to monitor antiretroviral treatment (ART) in Togo.

**Methods:**

Patients on HAART for one year (10-14 months) were enrolled between April and October 2008 at three sites in Lomé, the capital city of Togo. Plasma viral load was measured with the NucliSENS EasyQ HIV-1 assay (Biomérieux, Lyon, France) and/or a Generic viral load assay (Biocentric, Bandol, France). Genotypic drug-resistance testing was performed with an inhouse assay on plasma samples from patients with viral loads of more than 1000 copies/ml. CD4 cell counts and demographic data were also obtained from medical records.

**Results:**

A total of 188 patients receiving first-line antiretroviral treatment were enrolled, and 58 (30.8%) of them experienced virologic failure. Drug-resistance mutations were present in 46 patients, corresponding to 24.5% of all patients enrolled in the study. All 46 patients were resistant to non-nucleoside reverse-transcriptase inhibitors (NNRTIs): of these, 12 were resistant only to NNRTIs, 25 to NNRTIs and lamivudine/emtricitabine, and eight to all three drugs of their ARV regimes. Importantly, eight patients were already predicted to be resistant to etravirine, the new NNRTI, and three patients harboured the K65R mutation, inducing major resistance to tenofovir.

**Conclusions:**

In Togo, efforts to provide access to ARV therapy for infected persons have increased since 2003, and scaling up of ART started in 2007. The high number of resistant strains observed in Togo shows clearly that the emergence of HIV drug resistance is of increasing concern in countries where ART is now widely used, and can compromise the long-term success of first- and second-line ART.

## Background

Implementation of antiretroviral therapy (ART) is recognized as a public health priority in resource-limited countries. In order to allow a rapid roll out of ART, countries use the World Health Organization (WHO) public health approach, which proposes standard first-line therapy, together with treatment initiation and switch guided by clinical disease progression and, where possible, with monitoring of CD4 cell counts [1]. The standard therapy consists of two nucleoside reverse transcriptase inhibitors (NRTIs) (3TC+AZT/d4T) and one non-nucleoside reverse transcriptase inhibitor (NRRTI) (EFV/NVP).

In 2010, these guidelines were revised and recommended less toxic drugs in first-line therapy by replacing stavudine (d4T) with tenofovir (TDF) [2]. Although a more strategic monitoring for ART efficacy is now also recommended, virological monitoring is still not feasible for the majority of patients on ART in sub-Saharan Africa due to the absence of adequate laboratory facilities and insufficient financial means. In addition, deficiencies in health systems and resources, such as unreliable supply systems, storage and the lack of qualified personnel to prescribe and monitor patients on ART, could also create conditions for accelerated development of HIV resistance to antiretroviral (ARV) drugs. It is thus important to evaluate the outcome and effectiveness of ART programmes in routine care settings in resource-limited countries to evaluate whether the empirical second-line treatment recommended by WHO would still be effective.

Togo is a small country of 5.5 million inhabitants, located in west Africa, with an estimated HIV prevalence of around 3% in the general population [3]. Scaling up of ART started in 2007, and approximately 7000 HIV-1-infected individuals were receiving ART by the end of 2007. Treatment became free of charge by the end of 2008 and, today, more than 17,000 people are receiving ART, which corresponds to coverage of 33%. Here we describe virological outcome and emergence of drug resistance in a cross-sectional study among HIV-1-infected patients treated according to the national guidelines in hospitals in Lomé, the capital city of Togo.

## Methods

A total of 188 HIV-1-positive patients receiving first-line ART for 12 months (+/-2 months) were consecutively enrolled between April and October 2008 in three sites in Lomé: the University Hospital Tokoin; and two non-governmental organizations for HIV care, (EVT (Espoir Vie Togo) and CRIPS (Centre de Réflexion et d'Initiative pour la Promotion de la Santé). The study was approved by the National Ethics Comitee and Ministry of Health (N°0269/2007/MS/DGS/DPLET/CBRS). Only patients who declared that they were ARV treatment naïve prior to the start of first-line treatment and those without prior use of ARVs for prevention of mother to child transmission of HIV were included in this study. After written informed consent, whole blood was collected and plasma was separated from cells by centrifugation at 3000rpm for five minutes and stored at -80°C in three aliquots.

Whole blood less than six hours old was used to determine the CD4 lymphocyte counts using a FACSCALIBUR flow cytometer (Becton Dickinson, San Jose, CA). Plasma viral load was measured with the NucliSENS EasyQ HIV-1 assay (Biomérieux, Lyon, France) and/or a Generic viral load assay (Biocentric, Bandol, France) [4]; the detection limits of the tests are 50 and 300 copies/ml, respectively. Genotypic drug-resistance testing was performed on samples with HIV-1 RNA levels equal to or above 1000 copies/ml using a previously described in-house assay [5]. Amino acid sequences were analyzed for the presence of mutations in protease and RT genes with the drug-resistance interpretation algorithm from ANRS (version July 2010) (http://www.hivfrenchresistance.org/). HIV-1 subtypes/CRFs were determined by phylogenetic tree and recombination analysis as previously decribed [6].

## Results

During the study period, 580 HIV-1-infected patients attended one of the three clincs for their follow-up visit at M12, and a total of 188 were included in this study. The median duration of ARV therapy prior to study enrollment was 12 months, ranging from 10 to 14 months. The median age of patients was 37 years (IQR 32-43), and only 66 (35.1%) were male (Table 1). National guidelines for patient monitoring recommend monthly clinical visits and CD4 counts at start and every six months. CD4 counts were available for 160 patients at treatment initiation; a median CD4 cell count of 100 cells/mm^3 ^(IQR 54-173) was seen, which increased to a median of 293 cells/mm^3 ^(IQR 188-431) among the same 160 patients at the time of enrolment in this study, i.e., about 12 months later.

**Table 1:** Patient characteristics and HIV-1 variants

Characteristic	Patients (n = 188) Number (%)
Age, median years	37 (IQR 32-43)
**Male sex**	66 (35.1)
**Treatment interruption (days)**	
[1-30]	82 (43.6)
>30	22 (11.7)
Total	104 (55.3)
**CD4 lymphocyte at enrolment **(n = 160)	
CD4 count, median cells/mm^3^	100 (IQR 54-173)
**CD4 lymphocyte at end point **(n = 160)	
CD4 count, median cells/mm^3^	293 (IQR 188-431)
**Viral load at end point **≥1000 copies/ml	58 (30.9)
**Genotypic drug-resistance mutations**	46 (24.5)
HIV subtypes/CRFs	
CRF02	26
CRF06	6
G	1
Unique recombinant forms^a^	13

^a^.The following unique recombinant forms were observed: CRF02/A3 (n = 2), CRF02/CRF06 (n = 1), CRF02/U (n = 2), G/A (n = 1), G/A3 (n = 1), G/A/CRF02 (n = 1), G/CRF06 (n = 1), G/CRF02 (n = 2), CRF02/CRF06/U (n = 1), J/K/U (n = 1); U = Unknown subtype/CRF

However, CD4 count increases were lower for the patients with virological failure, i.e., from 98 cells/mm^3 ^(IQR 97-154) to 121 cells/mm^3 ^(IQR 50-249) versus 112 cells/mm^3 ^(IQR 54-176) to 347 cells/mm^3 ^(IQR 269-475). With the exception of two patients, all received the generic drug, Triomune, a fixed-dose combination of stavudine (d4T), lamivudine (3TC) and nevirapine (NVP). The other two patients received AZT+3TC+EFV or AZT+3TC+IDV. Importantly, during the 12-month ART period, 104 (52.7%) patients had interrupted their treatment; the duration of this interruption ranged from one to 168 days, and for 22 patients, this period exceeded one month. Treatment interruption was reported for patients with and without virological failure at comparable rates.

For 58 (30.8%) patients, plasma viral load (VL) was above 1000 copies/ml, and for nine of them, VL was between 1000 and 5000 copies/ml. Genotypic drug-resistance testing analyses were done on 56 samples for which sufficient material was available. Mutations conferring resistance to NRTIs were detected in 46 of 56 (82.2%) patients with VLs above 1000 copies/ml, including seven of the nine patients with VLs between 1000 and 5000 copies/ml. This corresponds to at least 24.5% of patients receiving ART for 12 months.

Details of the drug-resistance profiles and corresponding patient characteristics are shown in Table 2. All patients were resistant to NNRTIs: 12 were resistant only to NNRTIs, with the remainder resistant to NNRTIs and other drugs. Among the NNRTI-associated resistance mutations, 29 (60.4%) occurred at position Y181, 17 (35.4%) at K103 and 10 (20.8%) at G190 (Table 2). V106A/M, K101E and Y188C/L were noted in four, three and two patients, respectively. Importantly, eight patients were already predicted to be resistant to etravirine, the new NNRTI, either because they harboured the single Y181V mutation (n = 3) or due to the presence of both Y181C and H221Y mutations (n = 5). Among the 46 ARV-resistant patients, 25 also harboured the M184V mutation conferring resistance to 3TC/FTC; among them, eight patients were also resistant to the other NRTI drug in their regimen because of the high number of TAMs (n = 4), or the presence of the Q151M (n = 1) or the K65R (n = 3) mutation. This implied that they were resistant to all three drugs of their ARV regimes.

**Table 2:** Details of observed drug mutations and prediction of resistance to ARVs according to ANRS algoritm (version July 2010) and corresponding patient characteristics, CD4 counts and viral load values

Patient	Age	Sex	**CD4 (cells/mm**^**3**^**)**		Viral	Drug-resistance Mutations			Predicition of high level resistance to following ARVs
ID			M0	M12	load	NRTI (ex 3TC/FTC)	3TC/FTC	NNRTI	
T3003	28	F	175	50	5.68	M41LM,T215NSTY,V75MV	M184V	Y181C	EFV/NVP; 3TC/FTC, D4T, AZT
T3008	32	F	70	36	5.57	K65R, (T69D)	-	K101E, Y181C, G190S	EFV/NVP; TDF
T3013	23	F	nt	507	4.75	T69N	-	K103N, Y181C	EFV/NVP
T3017	68	M	53	203	4.88	-	M184V	(A98AG), K103KN,	EFV/NVP; 3TC/FTC
T3029	39	M	21	145	6.34	-	M184V	K101E, G190A,	EFV/NVP; 3TC/FTC
T3031	36	M	100	21	4.20	M41L, T215Y, V75MV,	M184V	Y181C	EFV/NVP; 3TC/FTC, D4T, AZT
T3034	39	M	154	209	4.59	-	M184V	Y181C	EFV/NVP; 3TC/FTC
T3039	33	F	181	264	5.08	-	M184V	Y181V	ETV, EFV/NVP; 3TC/FTC
T3041	47	M	348	218	4.99	-	M184V	K101E, G190A	EFV/NVP; 3TC/FTC
T3042	35	F	16	17	5.23	T69N, Q151M	M184V	V90I, K103N, Y181C, G190A	EFV/NVP; 3TC/FTC, ABC, AZT, D4T, DDI,
T3043	37	F	276	255	4.89	-	M184V	K103N	EFV/NVP; 3TC/FTC
T3046	43	M	36	43	4.64	K70R, T215F, K219E	M184V	Y181C	EFV/NVP; 3TC/FTC, AZT, D4T
T3047	**32**	M	128	254	4.41	-	-	Y181C, H221HY	ETV, EFV/NVP
T3049	33	F	154	338	4.25	-	M184V	G190A	EFV/NVP; 3TC/FTC
T3061	24	F	184	410	3.43	-	M184V	V106A,	NVP; 3TC/FTC
T3067	37	F	143	264	3.32	T69S	M184V	K103N,	EFV/NVP; 3TC/FTC
T3069	40	M	143	163	3.72	T69D, K70R, K219Q	M184V	Y181C, G190A, H221Y	ETV, EFV/NVP; 3TC/FTC
T3071	40	F	80	282	3.43	T69N	M184V	Y181C	EFV/NVP; 3TC/FTC
T3073	45	M	168	58	4.08	-	-	K103N, Y181C	EFV, NVP
E5009	35	M	78	43	5.56	D67N, K65R	-	V106M, Y188C	EFV/NVP; TDF
E5011	50	M	102	148	3.32	-	M184V	Y181C	EFV/NVP; 3TC/FTC
E5013	32	F	57	112	3.20	T69N	M184V	Y181C	EFV/NVP; 3TC/FTC, DDI
E5016	33	F	173	399	4.20	-	-	Y181CY	EFV/NVP
E5017	47	M	nt	182	4.23	-	M184V	Y181V	ETV; 3TC/FTC
E5020	22	F	147	365	3.04	-	M184V	K103N	EFV/NVP; 3TC/FTC
E5024	42	M	78	197	4.41	-	M184V	(V90I), V106A,	EFV/NVP; 3TC/FTC
E5025	48	F	51	71	3.32	K65R	-	Y181C, K103N	EFV/NVP; TDF
E5027	29	F	35	84	5.08	-	-	K103KN	EFV/NVP
E5032	39	M	70	13	5.45	-	-	G190AG, K103N	EFV/NVP
E5043	46	M	27	93	3.11	-	M184V	Y181V, (A98S)	ETV; 3TC/FTC
E5044	60	F	nt	517	4.78	-	M184V	Y188L	EFV/NVP; 3TC/FTC
E5047	52	F	57	57	4.54	-	-	Y181C, K103N	EFV/NVP
E5053	35	M	99	66	5.74	(D67N, L210W)	-	Y181C, G190AG, (V90I)	EFV/NVP
E5059	41	M	118	40	4.59	(T69N )	-	Y181C	EFV/NVP
E5061	31	F	34	72	4.72	M41L	M184I,	Y181C, V90IV, A98AG, H221Y	ETV, EFV/NVP; 3TC/FTC
E5068	30	F	152	339	3.15	-	M184V	K103N, (A98G)	EFV/NVP; 3TC/FTC
C7003	34	M	98	288	4.30	-	M184V	V106A,	NVP; 3TC/FTC
C7004	38	F	185	540	4.45	-	M184V	K103N, Y181C, (V90IV)	EFV,NVP; 3TC/FTC
C7012	40	M	75	267	3.23	-	M184V	Y181C	EFV/NVP; 3TC/FTC
C7017	42	M	12	40	4.98	-	-	K103N	EFV/NVP
C7022	31	F	64	310	4.08	-	M184V	Y181C	EFV/NVP; 3TC/FTC
C7023	27	F	101	83	3.36	-	M184MV	K103N, (A98AG)	EFV/NVP; 3TC/FTC
C7024	20	F	35	84	4.00	T69D, D67G, K70R, K219Q	M184V	Y181C, G190A, H221Y	ETV, EFV/NVP; 3TC/FTC, AZT, DDI
C7028	33	M	72	12	4.36	-	-	Y181C, K103N	EFV/NVP
C7030	30	F	190	603	3.51	-	-	G190AG	EFV/NVP
C7044	55	F	nt	204	3.38	-	M184V	Y181C, H221Y	ETV, EFV/NVP; 3TC/FTC

Viral load is expressed as log_10 _copies/ml. The abbreviations are for the following drugs: AZT - zidovudine; 3TC - lamivudine; FTC - emtricitabine; ddI - didanosine; d4T - stavudine; ABC - abacavir; TDF - tenofovir; EFV - efavirenz; NVP - nevirapine; ETV - etravirine

The presence of the K65R also means that tenofovir (TDF) will not be effective when used in the second-line regime, and the Q151M mutation commonly confers multi-drug resistance to NRTIs (AZT, ABC, ddI and d4T). In seven patients, the presence of one or two NRTI mutations (M41L, D67N) was also seen, but this had not yet resulted in drug resistance. Two patients had virus mutations indicative of the TAM-1 profile (M41L, L210W, T215S), and two of the TAM-2 profile (D67N, K70R, T215F, K219Q/R/E). No major mutations were seen in the protease gene.

The predominant HIV-1 variant in this study population was CRF02_AG (n = 26, 56.5%). Other variants were CRF06_cpx (n = 6, 13%), subtype G (n = 1, 2.1%), and 13 (28.3%) were unique recombinant forms.

Scaling up highly active antiretroviral therapy (HAART) to achieve universal access is the current priority of WHO and the Joint United Nations Programme on HIV/AIDS (UNAIDS). Several cohort studies, using virological monitoring, have shown that ART treatment in resource-poor settings has efficacy rates similar to those reported for developed countries [7-12]. However, the few studies reporting on ART using the public health approach showed more contrasted results, with virological succes after 12 months ranging from 50% to more than 90% [13,14]. But among these patients with virological failure, a significant proportion can be due to adherence problems, as shown in a study in Cameroon, where only 25% of patients with VLs over 1000 copies/ml were resistant to ARVs after 12 months [15].

In contrast, in this study in Togo, more than 80% of patients with virological failure at M12 were already resistant to ARVs, corresponding to 24.5% of all the patients enrolled in this study. Moreover, since the first-line regimen included two drugs with low genetic barriers, the majority of patients with HIV harbouring drug-resistance mutations were resistant to two of the three drugs from their regimens (25 of 46, or 54.3%); eight of 46 (17.4%) were resistant to all drugs in their ARV treatments. A total of 11 (23.9%) patients already harboured mutations conferring resistance to drugs for second-line regimes, such as TDF (n = 3) or the new NNRTI, etravirine (n = 8).

This is in agreement with observations from a recent study in Malawi, which also showed clearly that when only clinical and CD4 count cell criteria are used to monitor first-line ART failure, extensive NRTI and NNRTI resistance emerges, with 23% patients having resistance profiles that compromise second-line ART [16]. Importantly, our estimates of drug resistance in treated patients in Togo are most likely minimal estimates because we used a cross-sectional approach and only patients who are still under treatment were studied. Our study does not provide any information on how many patients dropped out of care or died and how many of those patients harbour drug-resistance mutations. However, the observed information is useful for clinicians managing patients and can serve as an indicator of ARV programme efficiency in patients still on treatment.

In Togo, efforts to provide access to ARV therapy for infected persons have increased since 2003, and scaling up of ART started in 2007. However, during the scaling-up period, the national programme encountered problems with stock management involving ARV drug substitution with the same molecules, administered separately as individual pills instead of as fixed-dosed combinations, or even interruption of the treatment.

Overall, our study shows major problems with the ART programme in Togo during the first year of scaling up HAART in the country, but does not reflect the situation in Togo three years later, which could be different. In a previous study, we reported also that 10% of recently diagnosed ARV-naive HIV-1 positive patients in 2007 were infected with HIV strains that already harboured a drug-resistance mutation [6]. However, the possibility cannot be excluded that some of the patients included in this and our previous study [6] were not ART naïve and already harboured ARV-resistant strains because this was based on self-reported information by the patients.

## Conclusions

Our results show clearly that the emergence of HIV drug resistance is of increasing concern in countries where ART is now widely used, and can compromise long-term success in treatment outcomes. Since individual patient monitoring for viral load and drug resistance is not yet possible, the system that WHO (HIVRESNET) has established for the surveillance of transmitted drug resistance and the monitoring of ART resistance at sentinel sites should be implemented in order to inform health authorities on the efficiency of first- and second-line ART and allow recommendations on future ART strategies [17]. The high number of resistant strains observed in Togo among ARV-naïve patients and patients on ART at the onset of ART scaling up in the country needs further attention, and additional studies are needed to evaluate actual drug-resistance rates.

## Competing interests

The authors declare that they have no competing interests.

## Authors' contributions

AYD and NV carried out the viral load assays, genotypic drug-resistance test and phylogentic analysis. MM organized quality assurance for viral load between different sites. AM, AP, KO and MS enrolled patients and collected data on patient history. PP, AS, AYD, MPD, MP and ED participated in the design of the study. AYD, MPD and PP coordinated the study. AYD, NV, ED and MP drafted the manuscript. All authors read and approved the final manuscript.
